# Anti-Biofilm Coatings Based on Chitosan and Lysozyme Functionalized Magnetite Nanoparticles

**DOI:** 10.3390/antibiotics10101269

**Published:** 2021-10-19

**Authors:** Vera Alexandra Spirescu, Adelina-Gabriela Niculescu, Ștefan Slave, Alexandra Cătalina Bîrcă, Gabriela Dorcioman, Valentina Grumezescu, Alina Maria Holban, Ovidiu-Cristian Oprea, Bogdan Ștefan Vasile, Alexandru Mihai Grumezescu, Ionela Cristina Nica, Miruna Silvia Stan, Ecaterina Andronescu

**Affiliations:** 1 Department of Science and Engineering of Oxide Materials and Nanomaterials, University Politehnica of Bucharest, 011061 Bucharest, Romania; veraspirescu@stud.fim.upb.ro (V.A.S.); adelina.niculescu@stud.fils.upb.ro (A.-G.N.); stefan.slave@gmail.com (Ș.S.); alexandra.birca@upb.ro (A.C.B.); bogdan.vasile@upb.ro (B.Ș.V.); ecaterina.andronescu@upb.ro (E.A.); 2 Lasers Department, National Institute for Lasers, Plasma and Radiation Physics, 077125 Magurele, Romania; gabriela.dorcioman@inflpr.ro (G.D.); valentina.grumezescu@inflpr.ro (V.G.); 3 Department of Microbiology and Immunology, Faculty of Biology, University of Bucharest, 077206 Bucharest, Romania; alina.m.holban@bio.unibuc.ro; 4 Department of Inorganic Chemistry, Physical Chemistry and Electrochemistry, Faculty of Applied Chemistry and Materials Science, Politehnica University of Bucharest, 011061 Bucharest, Romania; ovidiu.oprea@upb.ro; 5 Research Institute of the University of Bucharest—ICUB, University of Bucharest, 050657 Bucharest, Romania; 6 Academy of Romanian Scientists, Ilfov No. 3, 50044 Bucharest, Romania; cristina.nica@drd.unibuc.ro (I.C.N.); miruna.stan@bio.unibuc.ro (M.S.S.); 7 Department of Biochemistry and Molecular Biology, Faculty of Biology, University of Bucharest, 050095 Bucharest, Romania

**Keywords:** laser processing, lysozyme, magnetite-based coatings, nanostructured bioactive coatings, antimicrobial properties, antibiofilm activity

## Abstract

Biofilms represent a common and increasingly challenging problem in healthcare practices worldwide, producing persistent and difficult to manage infections. Researchers have started developing antibiotic-free treatment alternatives in order to decrease the risk of resistant microbial strain selection and for the efficient management of antibiotic tolerant biofilm infections. The present study reports the fabrication and characterization of magnetite-based nanostructured coatings for producing biofilm-resistant surfaces. Specifically, magnetite nanoparticles (Fe_3_O_4_) were functionalized with chitosan (CS) and were blended with lysozyme (LyZ) and were deposited using the matrix-assisted pulsed laser evaporation (MAPLE) technique. A variety of characterization techniques were employed to investigate the physicochemical properties of both nanoparticles and nanocoatings. The biological characterization of the coatings assessed through cell viability and antimicrobial tests showed biocompatibility on osteoblasts as well as antiadhesive and antibiofilm activity against both Gram-negative and Gram-positive bacterial strains and no cytotoxic effect against human-cultured diploid cells.

## 1. Introduction

As revealed by the National Institutes of Health (NIH), 65% of all microbial infections and 80% of all chronic infections are associated with biofilm formation [1]. Thus, biofilms retain a relevant impact on public health, increasing hospital costs and resulting in significant morbidity and mortality [2,3,4].

Specifically, biofilms represent multicellular, surface-associated communities of microorganisms that self-produce extracellular polymeric substances (EPS) that mainly consist of polysaccharides, extracellular DNA, and proteins [3,5,6]. The polymeric matrix offers protection and enhanced survival abilities, shielding them from the host’s immune system, hindering the diffusion of antimicrobial agents to the biofilm, and leading to poor treatment outcomes [7,8,9]. Moreover, biofilm-protected bacteria can be released, which is conducive to the appearance of new infection sites [7].

The burden of biofilm development must be especially considered for indwelling and implanted medical devices, including catheters, mechanical heart valves, pacemakers, stents, prosthetic joints and implants, voice prosthesis, and internal and external fixation devices [1,2,3,8,10]. The conventional approach for treating device-associated infections consists of the prophylactic administration of systemic antibiotics and debridement [10,11]. However, bacterial cells in biofilms can exhibit a 1000-fold or greater increase in antibiotic resistance compared to planktonic cells, thus limiting the efficiency of classic therapies [3,5,12]. Moreover, the inappropriate prescription and inadequate administration of antimicrobial therapeutics may lead to side effects, organ toxicity, and ever-increasing antibiotic resistance [10,13,14,15].

Because biofilm-related infections are very difficult to eradicate, the recent research focus was shifted towards preventing biofilm formation [16,17]. In particular, modification of the surface nanotopography of biomedical devices represents a promising strategy against microbial adhesion [18]. By incorporating antimicrobial nanocompounds within or on the surface of materials or by coating the implants with a bioactive nanostructured film, the surface can be optimized towards impeding microbial adhesion or destroying pathogens after their attachment [7,11,14,19,20]. Nanomaterials are currently being investigated for numerous biomedical applications, including diagnosis and therapy [21,22], and are considered to be a versatile and innovative strategy for the management of infectious diseases [23].

The high potential in many applications of iron oxide nanoparticles is the result of the combination of their magnetic properties with biocompatibility, reactive surface, stability, and so on. Based on their unique properties, iron oxide nanoparticles have attracted considerable interest in the last decade [24,25,26]. Among them, Fe_3_O_4_ is one of the most popular types of currently researched nanomaterials, especially due to its special magnetic properties, availability, versatility, eco-friendliness, and low cost. Moreover, their small size, excellent biocompatibility, biodegradability, non-toxicity to humans, and possibility for functionalization these bioactive magnetic make these nanostructures recommended for the development of unconventional antimicrobials [27,28,29,30,31]. Nevertheless, the properties of Fe_3_O_4_ nanoparticles depend on their preparation method, and many synthesis techniques have been employed to obtain optimal characteristics for different end purposes. Methods, such as co-precipitation, thermal decomposition, sol-gel, microemulsion, hydrothermal, sonochemical, electrochemical, and biological synthesis have been shown to successfully produce Fe_3_O_4_ nanostructures [24,28,32,33,34]. One of the simplest and most widely used chemical methods for obtaining nanosized Fe_3_O_4_ is co-precipitation [35,36,37], particularly due to its simplicity, high yields, and potential for reduced time-consuming, making it easily scalable in industrial applications [37]. Moreover, particle properties can be tuned by carefully adjusting the ratio of iron salts and the pH of the reaction medium [23,38,39].

Nonetheless, Fe_3_O_4_ nanoparticles are not stable in air, having a tendency to oxidize to maghemite, and can easily agglomerate after production. To avoid these drawbacks, Fe_3_O_4_ nanoparticles for biomedical purposes are usually protected by shells of different biocompatible materials, such as natural polysaccharides, inert synthetic materials, and organic acids with different structures [27,28,40]. From the plethora of materials that can be used to modify the surface of Fe_3_O_4_ nanoparticles, chitosan is one of the most attractive options.

Chitosan is a partially deacetylated linear polysaccharide of chitin [41]. Its natural origin and convenient biochemical properties (e.g., good tolerability, non-toxicity, good biocompatibility, proper biodegradation rate, antioxidant activity, antimicrobial activity) make this cationic polymer recommended for various biomedical applications [24,42,43,44,45,46,47,48,49,50,51,52]. Lysozyme is an important antibacterial component, catalytically hydrolyzing β (1→4) glycosidic linkages at the C_4_ atom within the N-acetyl-D-glucosamine units present in chitosan. This catalytic hydrolysis justifies the antibacterial nature of lysozyme, as it selectively degrades the cell walls of microorganisms without destroying other tissues [53,54]. More recently, several studies have investigated the effects of chitosan–lysozyme conjugates on bacterial strains [55,56].

In this context, we report the fabrication of novel biocompatible coatings with inhibitory activity against microbial biofilm formation based on Fe_3_O_4_ nanoparticles functionalized with chitosan and lysozyme by MAPLE (matrix-assisted pulsed laser evaporation) technique. We have selected this laser processing method because of its versatility, ability to obtain thin and uniform bioactive coatings, and the fact that it allows the deposition of virtually any type of chemical target while maintaining the properties of all the involved bioactives during processing [11,14]. The obtained nanocomposites were investigated from the compositional, morphological, and biological points of view by employing X-ray diffraction (XRD), thermogravimetric analysis with differential scanning calorimetry (TGA-DSC), scanning electron microscopy (SEM), transmission electron microscopy with selected area electron diffraction (TEM-SAED), Fourier-transform infrared spectroscopy (FT-IR), infrared microscopy (IRM), cell viability, and antimicrobial tests. Results have shown that the obtained thin nanostructured coatings could be considered for the future development of antibiofilm surfaces, showing great potential in prosthetics and regenerative medicine.

## 2. Results and Discussions

### 2.1. Physicochemical Investigation of Fe_3_O_4_@CS Nanoparticles

**Figure 1:**
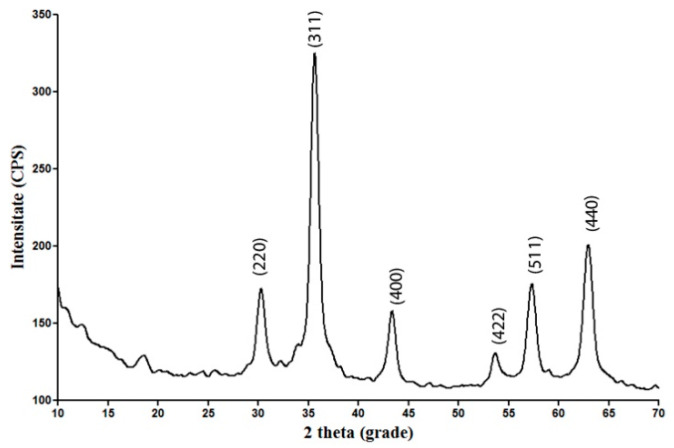
X-ray diffractogram of Fe_3_O_4_@CS nanoparticles.

The XRD pattern of the Fe_3_O_4_@CS nanoparticles is presented in Figure 1. The strong diffraction peaks appearing at 2θ diffraction angles of 30.0°, 35.4°, 44.0°, 53.4°, 56.9°, and 63.5° correspond to the diffraction planes (2 2 0), (3 1 1), (4 0 0), (4 2 2), (5 1 1), and (4 4 0), respectively, which is characteristic for crystalline magnetite with a spinel cubic structure. The strongest peak in the diffractogram is identified for a 2θ angle of 30°. All of the peaks are in agreement with the standard spectrum of Fe_3_O_4_ (DB card No. 9006242).

**Figure 2:**
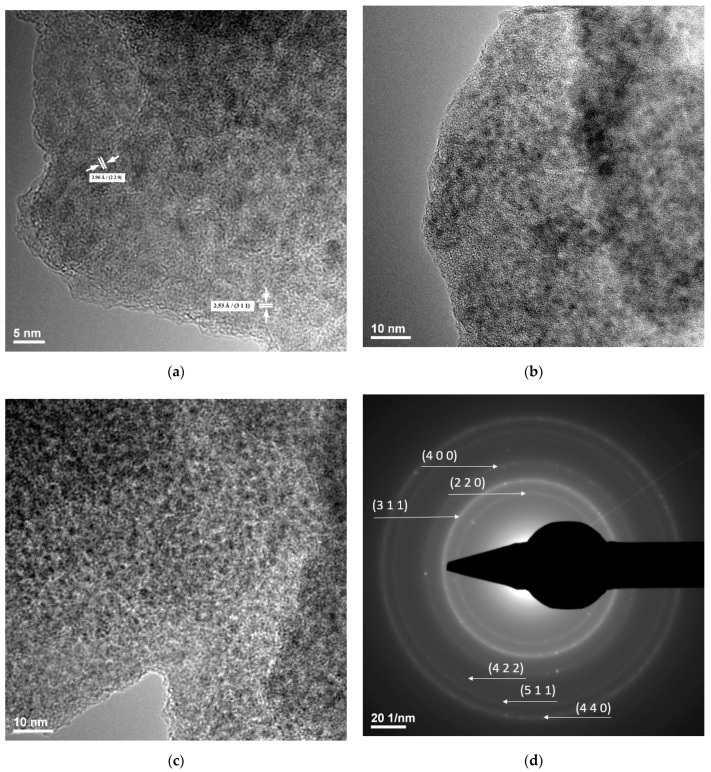
TEM images (**a**–**c**) and SAED pattern (**d**) of Fe_3_O_4_@CS nanoparticles.

The TEM investigation gathered relevant information on the distribution and composition of the crystalline phase in the samples of the Fe_3_O_4_@CS particles (Figure 2). From the micrographs recorded at 20 nm and 10 nm, the homogeneous distribution of the magnetite nanoparticles embedded in the chitosan matrix can be observed. Furthermore, TEM images confirm that the dimension of the particles is at the nanoscale, showing their organization in areas where the particles are dispersed.

The SAED pattern of the concentric diffraction rings formed at 220, 311, 400, 422, 511, and 440 are in excellent agreement with the results of the XRD analysis, thus confirming the crystalline nature of the prepared magnetite (Figure 2d).

**Figure 3:**
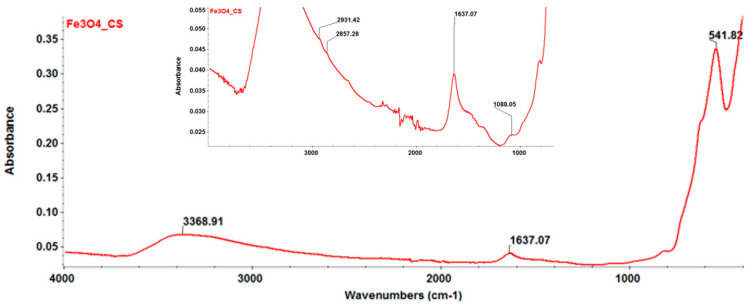
FT-IR spectrum of Fe_3_O_4_@CS nanoparticles.

The FT-IR analysis highlighted the integrity of the main functional groups of the prepared Fe_3_O_4_@CS nanocomposite (Figure 3). The absorption band recorded at 541 cm^−1^ corresponds to the Fe–O stretching vibrations from the structure of the magnetite; the absorption bands between 1088 and 3368 cm^−1^ are generated by the functional bonds from the structure of the chitosan, namely C-O (1088 cm^−1^), C=O (1637 cm^−1^), and C-H (2857 cm^−1^ and 2931 cm^−1^). The absorption band that is characteristic to the hydroxyl and amino groups is observed at 3368 cm^−1^.

**Figure 4:**
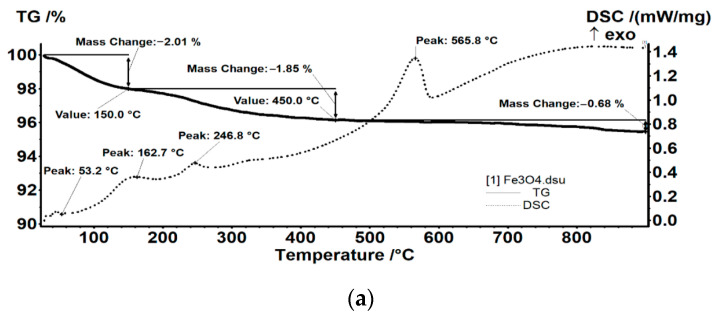

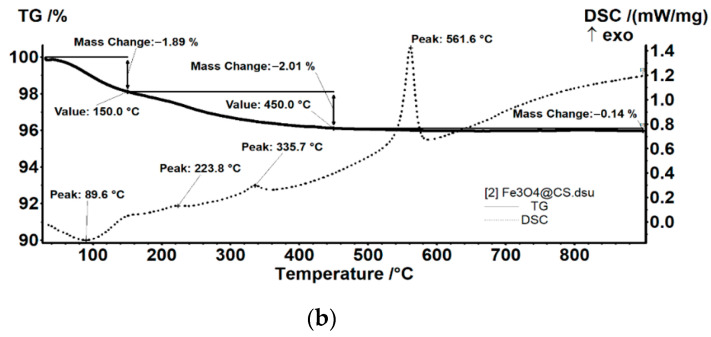
Thermogravimetric analysis of (**a**) Fe_3_O_4_ and (**b**) Fe_3_O_4_@CS nanoparticles.

A thermal analysis was realized on pristine and Fe_3_O_4_@CS (Figure 4). From Figure 4b, it can be seen that in the interval of RT-150 °C, the Fe_3_O_4_@CS sample loses 1.89% of its initial mass. The process is accompanied by an endothermic effect on the DSC curve, with the minimum at 89.6 °C. This mass loss can be assigned to the elimination of water molecules from the chitosan matrix and the surface of the nanoparticles. In the 150–450 °C interval, the sample loses 2.01% of its mass, which is probably due to the elimination of the –OH moieties from the nanoparticle surface but is also probably due to the oxidative degradation of the organic parts [57]. The weak exothermic effect from 223.8 °C can be assigned to the oxidation of Fe^2+^ to Fe^3+^ (transformation of magnetite to maghemite). The exothermic effect from 335.7 °C can be attributed to the oxidation of the chitosan [58]. After 450 °C, the sample loses 0.14% of its initial mass. The intense exothermic effect from 561.6 °C is due to the phase transformation of maghemite to hematite [14,59]. This thermal behavior pattern is similar to the one of the bare Fe_3_O_4_ particles (Figure 4a). 

### 2.2. Physicochemical Investigation of the Coatings

**Figure 5:**
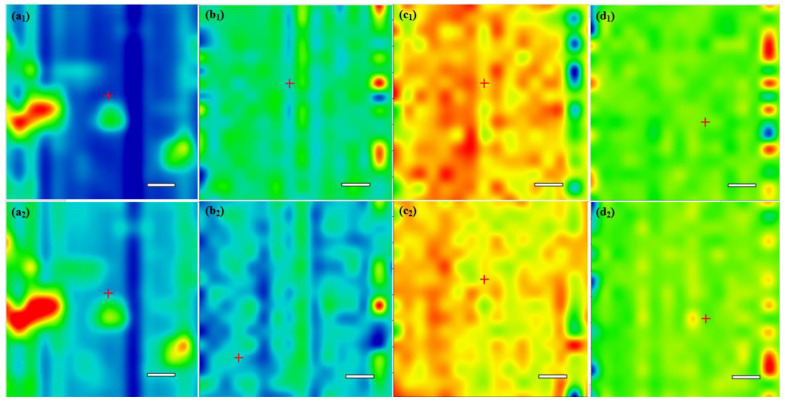
IRM of Fe_3_O_4_@CS drop-cast (**a**) and Fe_3_O_4_@CS thin coatings at 300 mJ/cm^2^ (**b**), 400 mJ/cm^2^ (**c**), and 500 mJ/cm^2^ (**d**) based on the distribution of C-H (1) and C-N (2) bonds from CS; scale bar 100 µm.

The thin coatings were characterized using IRM analysis. In this respect, IR maps were recorded for the Fe_3_O_4_@CS drop-cast (Figure 5a), and coatings were obtained at the 300, 400, and 500 mJ/cm^2^ laser fluences (Figure 5b–d) to allow a comparative analysis of the chemical distribution. The absorbance intensities of the IR spectra maps are proportional to color changes starting with blue (the lowest intensity) and gradually increasing through green, yellow, to finally red (the highest intensity). Thus, by comparing the IRM, it can be observed that the lowest functional group degradation was recorded at the 400 mJ/cm^2^ laser fluence. At this fluence, the color distribution is better than that of the other two laser fluences, showing that the thin coatings were deposited in the most homogeneous and uniform layer.

**Figure 6:**
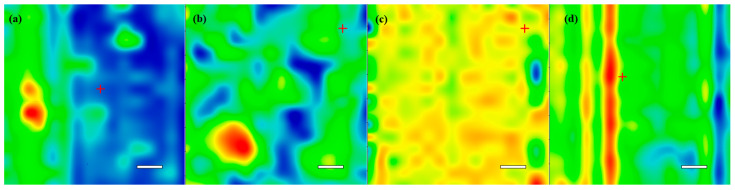
IRM of Fe_3_O_4_@CS/LyZ drop-cast (**a**) and Fe_3_O_4_@CS/LyZ thin coatings at 300 mJ/cm^2^ (**b**), 400 mJ/cm^2^ (**c**), and 500 mJ/cm^2^ (**d**) based on the distribution of C-N bonds from CS and LyZ; scale bar 100 µm.

Similar observations were made by comparing the IRM of the Fe_3_O_4_@CS/LyZ coatings (Figure 6).

**Figure 7:**
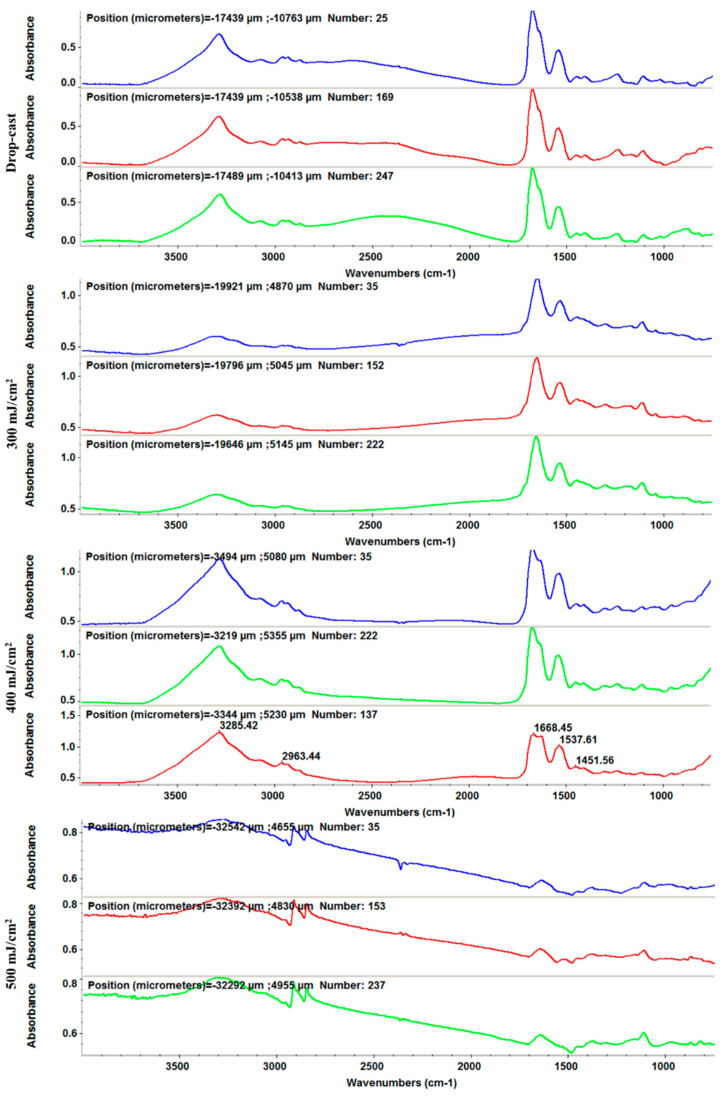
IR spectra of Fe_3_O_4_@CS/LyZ drop-cast and coatings obtained at differences laser fluence.

Complementary information was provided by analyzing the IR spectra of the Fe_3_O_4_@CS (data not shown) and the Fe_3_O_4_@CS/LyZ drop-cast and coatings obtained at 300, 400, and 500 mJ/cm^2^. The Fe_3_O_4_@CS and Fe_3_O_4_@CS/LyZ coatings recorded the lowest degree of functional group degradation at 400 mJ/cm^2^ laser fluence (Figure 7). For the other two laser fluences at which the magnetite-based layers were deposited, modifications in the intensities of the absorption bands can be observed. The decreases in the absorbance maxima, compared to drop-cast spectra, can be attributed to an insufficient transfer of the composite materials. In contrast, the loss and position shifting of some infrared maxima indicates that the laser beam damaged the chemical structure of the transferred material. 

Analyzing the integrity of IR spectra for each sample deposited at different laser fluences and the corresponding drop-cast coating, we selected the 400 mJ/cm^2^ laser fluence value as the best compromise between the deposition rate and the stoichiometric transfer to deposit the composite coatings for biological assays.

**Figure 8:**
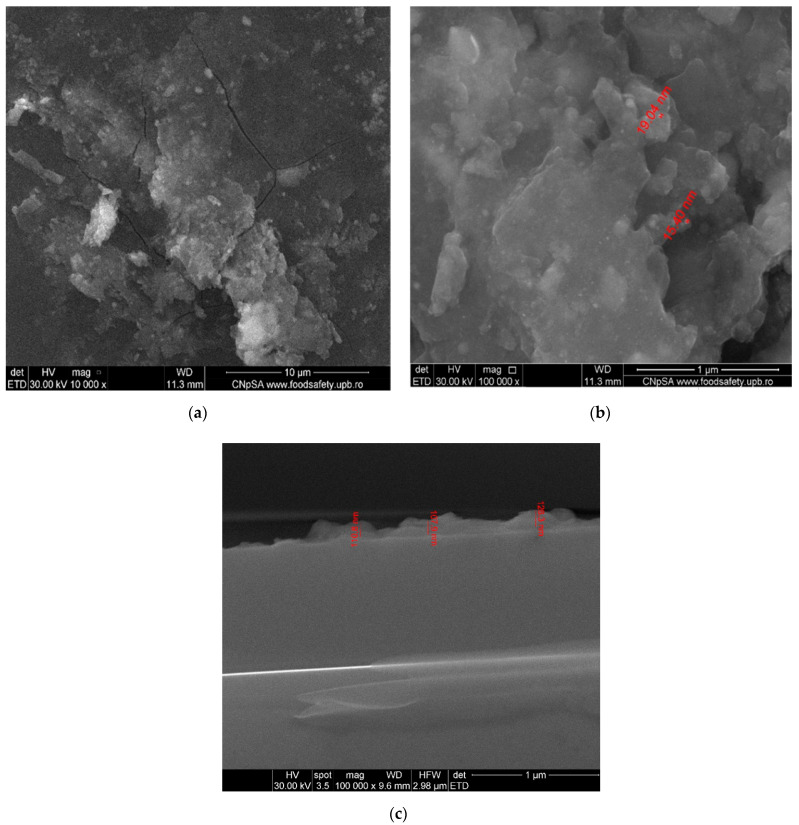
SEM micrographs of Fe_3_O_4_@CS/LyZ coating: (**a**,**b**) surface; (**c**) cross-section.

The thin coatings deposited at the 400 mJ/cm^2^ laser fluence were analyzed by SEM (Figure 8). It can be seen that the thin coatings contain higher numbers of aggregates on the top of their surfaces, with diameters between 20 and 50 nm. The surface is completely covered. Several artificial cracks were induced before SEM analysis in order to highlight this. Cross-section analysis highlights a thickness of 100–120 nm.

### 2.3. Biological Evaluation of the Coatings

#### 2.3.1. Cell Viability

**Figure 9:**
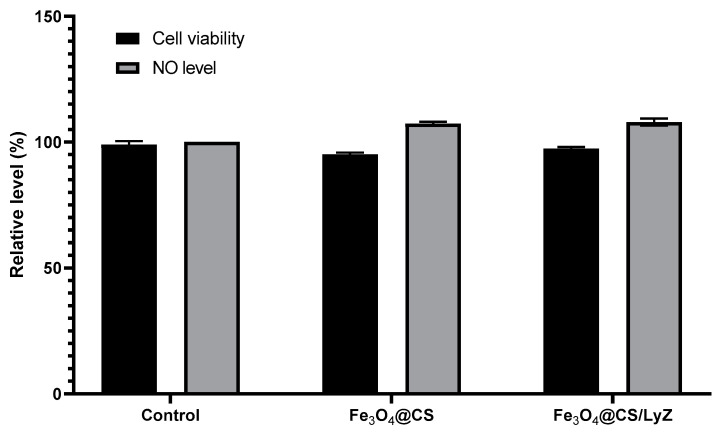
Cell viability and NO level after 24 h-incubation of MC3T3-E1 osteoblasts with Fe_3_O_4_@CS and Fe_3_O_4_@CS/LyZ samples. The results were calculated as mean values (*n* = 3) and expressed relative to control samples (uncoated substrate).

**Figure 10:**
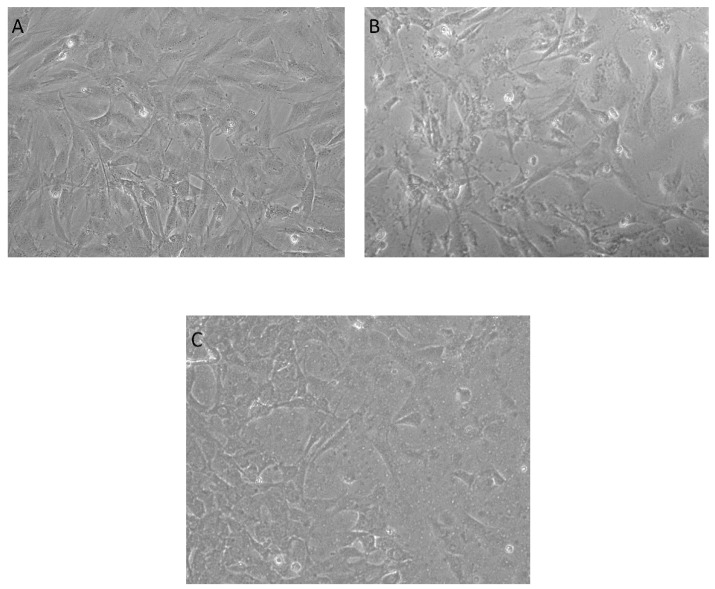
Phase-contrast microscopy images of osteoblasts grown for 24 h on (**A**) control, (**B**) Fe_3_O_4_@CS coatings, and (**C**) Fe_3_O_4_@CS/LyZ coatings (objective 10×).

For the biological characterization of the obtained coatings, the percentage of metabolically active cells was evaluated through an MTT (3-(4,5-dimethylthiazol-2-yl)-2,5-diphenyltetrazolium bromide) assay on murine osteoblasts (Figure 9). It can be noted that neither Fe_3_O_4_@CS nor Fe_3_O_4_@CS/LyZ showed cellular toxicity, as their cell viability percentages were ~95% and ~98% of the uncoated control, respectively. The Griess test performed to measure the nitric oxide (NO) level also led to favorable results. As it can be seen in Figure 10, the amount of NO released into the culture media was around ~107% of control for both types of coatings.

The cell viability was also confirmed by optical microscopy images (Figure 10), as the number of healthy cells in the presence of the coatings was comparable to the number of cells grown on the control sample.

#### 2.3.2. Antimicrobial Tests

*Staphylococcus aureus* (Gram-positive bacteria model) and *Pseudomonas aeruginosa* (Gram-negative bacteria model) bacteria represent two of the most infectious threats in the hospital environment because of their wide distribution, opportunistic behavior, and increasing antibiotic resistance. In this respect, the in vitro evaluation of bacterial biofilm anti-adherent properties of the prepared nanostructured coatings was assessed against both of these pathogens. 

**Figure 11:**
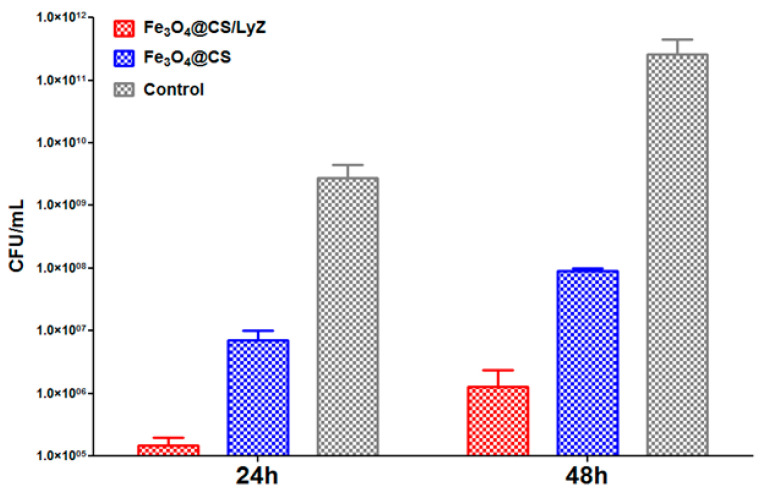
Evaluation of *S. aureus* microbial biofilm development after 24 and 48 h in the presence of the bioactive nanomodified coatings.

Figure 11 shows the antibiofilm results obtained for *S. aureus* at 24 and 48 h of incubation in the presence of the bioactive coatings. For this Gram-positive microorganism, a high CFU (colony forming units)/mL value was registered for the control sample (magnitude order of 1.0 × 10^9^ at 24 h and 1.0 × 10^11^ at 48 h). Compared to these values, the Fe_3_O_4_@CS and Fe_3_O_4_@CS/LyZ coatings showed much lower CFU/mL values (10^5^–10^7^), suggesting a low ability to develop biofilms on the analyzed coatings. The biofilm inhibition ranged from 1.5 up to 4 logs, depending on the analyzed sample. Specifically, the chitosan-modified magnetite obtained values to the order 1.0 × 10^7^ and 1.0 × 10^8^ CFU/mL after 24 and 48 h, respectively. For the Fe_3_O_4_@CS/LyZ sample, there were recorded values to the order of 1.0 × 10^5^ at 24 h and 1.0 × 10^6^ at 48 h, which are much better than both the control Fe_3_O_4_@CS samples. These results demonstrate the anti-adherent and antibiofilm character of both of the tested magnetite-based nanocomposites. However, the highest biofilm inhibition potential was observed for CS/LyZ containing the magnetite NP sample, suggesting a synergic antibacterial effect of Cs and LyZ (Figure 11).

**Figure 12:**
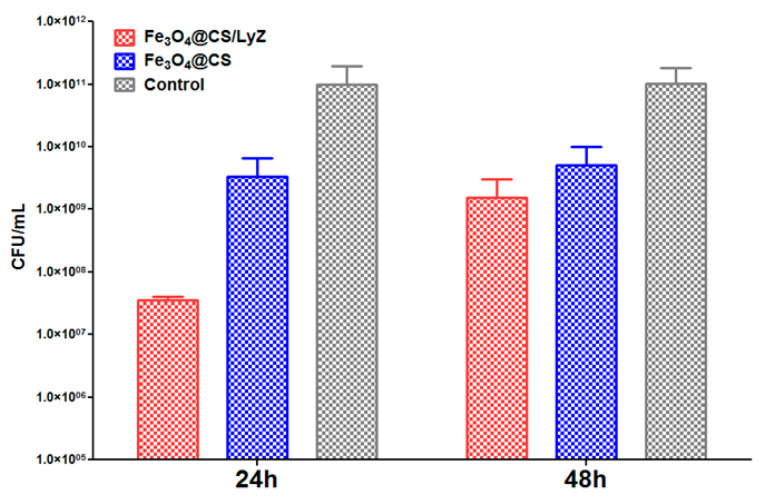
Evaluation of *P. aeruginosa* microbial biofilm development after 24 and 48 h in the presence of the bioactive nanomodified coatings.

In the case of *P. aeruginosa* strains (Figure 12), the control CFU/mL values are to the order of 1.0 × 10^11^ for both incubation periods. Compared to the control samples, the CFU/mL values for the chitosan-modified magnetite nanocomposite are significantly lower (2–4 logs), reaching values to the order of 1.0 × 10^9^ both at 24 and 48 h intervals. A considerable enhancement in the inhibitory character can be noted for the lysozyme-containing coating, sowing CFU/mL values of the 1.0 × 10^7^ order of magnitude after 24 h of incubation. However, at 48 h, the difference between Fe_3_O_4_@CS and Fe_3_O_4_@CS/LyZ slightly diminishes.

The obtained antimicrobial results suggest that the obtained coatings show biofilm inhibition potential for at least two days, which is very important for subsequent potential biomedical applications, such as medical implants and bioactive dressings.

## 3. Materials and Methods

### 3.1. Materials

The chemical substances required to synthesize the nanostructured materials, i.e., ferrous sulfate (FeSO_4_), ferric chloride (FeCl_3_), chitosan, lysozyme, acetic acid, ammonium hydroxide (NH_4_OH), dimethyl sulfoxide (DMSO), were purchased from Sigma Aldrich (Merck Group, Darmstadt, Germany). All chemicals were used without any further purification, and all solutions were prepared using ultrapure water (MiliQ^®^, Merck Millipore, Burlington, MA, USA).

### 3.2. Methods

#### 3.2.1. Synthesis of Fe_3_O_4_@CS

The Fe_3_O_4_ nanoparticles functionalized with CS were synthesized by the co-precipitation method, which involved the prior preparation of two solutions. The first solution contained the Fe precursors and was prepared by adding 1.6 g of FeSO_4_ and 1 g of FeCl_3_ into 300 mL of demineralized water. To this mixture, 100 mL of CS 1% was added. The second solution contained 9 mL of NH_4_OH and 300 mL of deionized water mixed under magnetic stirring. The precursor solution was added dropwise to the alkaline solution under continuous stirring. After decanting, the aqueous solution containing the reaction by-products was removed, and the powder was washed three times with deionized water. The final product was left to dry at room temperature. 

#### 3.2.2. MAPLE Target Preparation and Deposition of Composite Coatings

DMSO solutions of 1.5% Fe_3_O_4_@CS and Fe_3_O_4_@CS blended with LyZ (2:1 wt%) (Fe_3_O_4_@CS/LyZ) were prepared. All MAPLE targets were obtained by freezing the solutions poured into a pre-cooled holder at 173 K and were subsequently immersed in liquid nitrogen for 30 min. The substrates were successively cleaned into an ultrasonic bath with acetone, ethanol, and deionized water, and they were then plasma-cleaned into an oxygen atmosphere for 15 min with a plasma system (Diener electronic, GmbH). For comparison data, a control set of coatings was prepared by drop-cast on (1 0 0) silicon. MAPLE depositions were performed using a KrF* (λ = 248 nm and τ_FWHM_ = 25 ns) laser source COMPexPro 205 model (Lambda Physics-Coherent) operating at the repetition rate of 15 Hz. The laser fluence was set in the 300–500 mJ/cm^2^ range. All coatings were grown at a 4 cm target-substrate separation distance by applying (42,000–110,000) subsequent laser pulses. Thin coatings were deposited onto both sides of the polished (1 0 0) silicon and glass substrates for IRM, SEM, and biological assays. After physico-chemical analysis we selected the 400 mJ/cm^2^ fluence of the laser to be utilized in all of the subsequent biological tests.

#### 3.2.3. Physicochemical Characterization

##### XRD

The crystallinity of the obtained nanopowder was investigated by XRD using a Shimadzu XRD 6000 diffractometer. The XRD analysis was accomplished at room temperature at the Bragg diffraction angle range between 10 and 80° using CuK α radiation with λ = 1.056 Å (15 mA and 30 kV).

##### SEM

To investigate the morphology and dimensions of the nanostructured thin layers, the samples were sectioned using a diamond disc placed on a support and were introduced into an FEI (Hillsboro, OR, USA) electron microscope. The obtained images were recorded using secondary electron beams at an energy of 30 keV.

##### TEM

For TEM investigations, a small quantity of the sample powder was dispersed in pure ethanol and was subjected to an ultrasonic treatment for 15 min. The sample was placed on a carbon-copper grid and was left to dry at room temperature. The record the TEM micrographs, a Tecnai^TM^ G2 F30 S-TWIN high-resolution transmission electron microscope from FEI Company (Hillsboro, OR, USA) was used in the transmission mode at a 300 kV voltage with point and line resolutions of 2 Å and 1 Å, respectively. The apparatus’ selected area electron diffraction (SAED) accessory allowed the acquisition of additional crystallographic data.

##### FT-IR

To investigate the integrity of functional groups characteristic to synthesized particles, a reduced quantity of particle suspension was analyzed using a Nicolet 6700 FT-IR spectrometer from Thermo Fischer Scientific. The measurements were performed at room temperature, with 32 scans being collected at the 4000 and 1000 cm^−1^ range with a 4 cm^−1^ resolution. Recording the as-acquired information was possible by connecting the apparatus to a unity of data processing using Omnic Picta 8.2 software (Thermo Fischer Scientific). Thus, the collected spectra were overlapped, and the absorbance maps were created based on the second derivative of the spectral data.

##### TGA-DSC

The thermal analysis TGA-DSC for the precursors was performed with a Netzsch STA 449C Jupiter apparatus. The samples were placed in an open crucible made of alumina and were heated at 10 K·min^−1^ from room temperature up to 900 °C under the flow of 50 mL min^−1^ dried air. An empty alumina crucible was used as a reference.

#### 3.2.4. Biological Characterization

##### Cell Viability

To determine the cell viability of the nanocomposite films, an MTT viability assay was conducted on mouse osteoblasts MC3T3-E1 grown for 24 h in Minimum Essential Medium containing 10% fetal bovine serum. The investigated thin films deposited on Si substrates were previously UV sterilized by exposure for 20 min on each side). Cells were seeded on top of uncoated and coated substrates at a cellular density of 4 × 10^4^ cells/cm^2^. After removing the culture medium, the cells were washed with phosphate-buffered saline (PBS). The MTT solution was added, and the cells were further incubated at 37 °C for two hours in the dark. The MTT solution was removed and replaced with an equal volume of isopropanol to solubilize the formazan crystals thorough pipetting. The spectrophotometric absorbance measurements were performed at a 595 nm wavelength with the aid of a GENios TECAN microplate reader (TECAN, Männedorf, Switzerland). The cell morphology was visualized using an Olympus IX71 microscope (Olympus, Tokyo, Japan). Uncoated substrates were considered the control for the biological tests.

The amount of NO in the collected culture medium after the osteoblasts had been incubated with the test samples for 24 h was measured with Griess reagent (a stoichiometric solution of 0.1% naphthylethylenediamine dihydrochloride and 1% sulphanilamide in 5% H_3_PO_4_). Increased NO levels were significant for cytotoxic effects related to inflammation and apoptosis processes. The absorbance of the mix formed from culture supernatants and Griess reagent was measured at 550 nm using the GENios TECAN reader, and the NO concentration was calculated from the standard NaNO_2_ curve.

##### Antimicrobial Effect

To test the effect of the prepared surfaces on biofilm formation, the obtained materials were cut into 1 cm × 1 cm samples and were sterilized by UV exposure for 20 min on each side. Each sterile fragment was individually placed in wells of a 6-well plate. An amount of 2 mL of nutritive broth were added to each well followed by 50 μL of bacterial suspensions of 0.5 McFarland standard densities (1.5 × 10^8^ CFU (colony forming units)/mL). The as-prepared 6-well plates were incubated at 37 °C for 24 h. After incubation, the samples were washed with PBS, and the culture medium was changed to ensure microbial biofilm development. The plates were further incubated for 24 h; afterward, the specimen on which biofilm was formed was washed with PBS and was placed in an Eppendorf tube containing 1 mL PBS. The tube was vigorously vortexed for 30 s to detach the biofilm cells. The obtained cell suspension was serially diluted, and different dilutions were seeded on nutritive agar in triplicate to perform viable counts and to quantify the number of colony-forming units (CFU/mL).

Biological test results were analyzed using Student’s *t*-test on Excel (Microsoft Office 2018). Statistically significant data were considered as having a *p*-value of less than 0.05.

## 4. Conclusions

This study presented the successful preparation of a nanomaterial based on chitosan and lysozyme functionalized magnetite, which is intended for future study and application in the biomedical domain. The initial nanopowders were deposited as thin coatings using the MAPLE technique and were further investigated from physicochemical and biological points of view. The developed nanostructured coating proved to have good biocompatibility and biofilm inhibitory activity against relevant opportunistic bacteria known for their biofilm infections. It was concluded that Fe_3_O_4_@CS and Fe_3_O_4_@CS/LyZ prepared coatings have a strong antimicrobial effect while maintaining high cell viability. The higher antibiofilm effect of the Fe_3_O_4_@CS/LyZ coating could be explained by the synergic effects of CS and LyZ, which are both known antimicrobial agents. These results are promising for the use of the prepared materials as bioactive nanostructured coatings for medical implants, which can aid in the prevention and treatment of persistent infections caused by microbial biofilms.

## Data Availability

Not applicable.
